# Analysis of Metabolic and Quality-of-Life Factors in Patients With Cancer for a New Approach to Classifying Walking Habits: Secondary Analysis of a Randomized Controlled Trial

**DOI:** 10.2196/52694

**Published:** 2025-04-01

**Authors:** Yae Won Tak, Junetae Kim, Haekwon Chung, Sae Byul Lee, In Ja Park, Sei Won Lee, Min-Woo Jo, Jong Won Lee, Seunghee Baek, Yura Lee

**Affiliations:** 1 Department of Information Medicine Asan Medical Center University of Ulsan College of Medicine Seoul Republic of Korea; 2 Graduate School of Cancer Science and Policy National Cancer Center Gyeonggi-do Republic of Korea; 3 Swallaby Co, Ltd Seoul Republic of Korea; 4 Division of Breast Surgery, Department of Surgery Asan Medical Center University of Ulsan College of Medicine Ulsan Republic of Korea; 5 Devision of Colorectal Surgery, Department of Surgery Asan Medical Center University of Ulsan College of Medicine Seoul Republic of Korea; 6 Department of Pulmonary and Critical Care Medicine Asan Medical Center University of Ulsan College of Medicine Seoul Republic of Korea; 7 Asan Medical Institute of Convergence Science and Technology Asan Medical Center University of Ulsan College of Medicine Seoul Republic of Korea; 8 Department of Preventive Medicine University of Ulsan College of Medicine Seoul Republic of Korea; 9 Department of Clinical Epidemiology and Biostatistics Asan Medical Center University of Ulsan College of Medicine Seoul Republic of Korea

**Keywords:** telemedicine, mobile phone, physical activity, mobile apps, mobile health intervention, cancer, step count

## Abstract

**Background:**

As the number of people diagnosed with cancer continues to increase, self-management has become crucial for patients recovering from cancer surgery or undergoing chemotherapy. Technology has emerged as a key tool in supporting self-management, particularly through interventions that promote physical activity, which is important for improving health outcomes and quality of life for patients with cancer. Despite the growing availability of digital tools that facilitate physical activity tracking, high-level evidence of their long-term effectiveness remains limited.

**Objective:**

This study aimed to investigate the effect of long-term physical activity on patients with cancer by categorizing them into active and inactive groups based on step count time-series data using the mobile health intervention, the Walkon app (Swallaby Co, Ltd.).

**Methods:**

Patients with cancer who had previously used the Walkon app in a previous randomized controlled trial were chosen for this study. Walking step count data were acquired from the app users. Biometric measurements, including BMI, waist circumference, blood sugar levels, and body composition, along with quality of life (QOL) questionnaire responses (European Quality of Life 5 Dimensions 5 Level version and Health-related Quality of Life Instrument with 8 Items), were collected during both the baseline and 6-month follow-up at an outpatient clinic. To analyze step count patterns over time, the concept of sample entropy was used for patient clustering, distinguishing between the active walking group (AWG) and the inactive walking group (IWG). Statistical analysis was performed using the Shapiro-Wilk test for normality, with paired *t* tests for parametric data, Wilcoxon signed-rank tests for nonparametric data, and chi-square tests for categorical variables.

**Results:**

The proposed method effectively categorized the AWG (n=137) and IWG (n=75) based on step count trends, revealing significant differences in daily (4223 vs 5355), weekly (13,887 vs 40,247), and monthly (60,178 vs 174,405) step counts. Higher physical activity levels were observed in patients with breast cancer and younger individuals. In terms of biometric measurements, only waist circumference (*P*=.01) and visceral fat (*P=*.002) demonstrated a significant improvement exclusively within the AWG. Regarding QOL measurements, aspects such as energy (*P*=.01), work (*P*<.003), depression (*P*=.02), memory (*P*=.01), and happiness (*P*=.05) displayed significant improvements solely in the AWG.

**Conclusions:**

This study introduces a novel methodology for categorizing patients with cancer based on physical activity using step count data. Although significant improvements were noted in the AWG, particularly in QOL and specific physical metrics, differences in 6-month change between the AWG and IWG were statistically insignificant. These findings highlight the potential of digital interventions in improving outcomes for patients with cancer, contributing valuable insights into cancer care and self-management.

**Trial Registration:**

Clinical Research Information Service by Korea Centers for Diseases Control and Prevention, Republic of Korea KCT0005447; https://tinyurl.com/3zc7zvzz

## Introduction

By 2040, it is projected that there will be 28.4 million new cases of cancer worldwide, indicating a 47% increase compared with the 19.3 million cases reported in 2020 [1]. Consequently, enhancing the quality of life (QOL) for patients with cancer has become imperative owing to the rising number of patients with cancer and their extended life expectancy. In the past, cancer interventions primarily focused on conventional treatments such as surgery, chemotherapy, and radiation therapy, following standardized protocols and guidelines [2]. However, the field of cancer management has now recognized the significance of personalized and holistic care in enhancing QOL, reducing disability, and restoring functions [3-5]. Hence, it is crucial to assess the potential benefits of physical activity in the context of cancer [6,7].

As physical activity measures patients’ activeness and fitness, evidence is increasingly establishing its status as a new indicator, particularly due to its characteristic of assessing long-term trends rather than cross-sectional snapshots such as body weight [8-10]. In previous studies, reports of physical activity in patients with cancer were cross-sectional and subjective [5-7]. Regular exercise has shown superior long-term effects, together with interventions aimed at changing behavior [11-14]. However, behavior change cannot be measured by cross-sectional indicators [15]. Therefore, quantitative and object data analysis is necessary to investigate the effectiveness of long-term physical activity using mHealth interventions. At the same time, commercial smartphone apps have many limitations in research, especially in collecting physical activity data over a sufficient period [16]. Consequently, the practical application of the research results to patients with cancer in the real world is limited, although eHealth tools can provide a potent resource to facilitate personalized and accessible care in daily life [11,12].

This study aimed to investigate the effect of long-term physical activity on patients with cancer by categorizing them into active and inactive groups based on step count time-series data, using the mHealth intervention, the Walkon app (Swallaby Co, Ltd.). We analyzed the long-term effects of physical activity by comparing the active and inactive walking groups based on physical parameters (eg, BMI, waist circumference, blood sugar levels, and body composition) and subjective impact on quality of life (eg, EQ-5D-5L [European Quality of Life 5 Dimensions 5 Level version] and HINT-8 [Health-related Quality of Life Instrument with 8 Items]). This study addresses a significant gap in research by evaluating the effectiveness of a digital intervention on the physical and mental well-being of patients with cancer, thus making a valuable contribution to the field of cancer care and patient self-management.

## Methods

### Study Design

This research study is a secondary data analysis of data collected at the start of a randomized controlled trial called: “Promotion of better lifestyle with precise and practicable digital health care in postoperative patients with cancer through multidisciplinary network” [17-19], which aimed to compare the effectiveness of 4 different smartphone health care applications in improving the quality of life of patients with cancer. In this secondary analysis, we aimed to identify groups that achieved behavioral changes in physical activity targeted by the intervention, and to evaluate its effectiveness through time series analysis of app usage data (step counts) of one arm of study participants and pre- and postcomparison of outcome variables (body measurements, blood tests, and QoL questionnaires). The primary study was registered with the Clinical Research Information Service of the Korea Center for Disease Control and Prevention, Republic of Korea (KCT0005447) [20].

### Data Collection

The step count data measured from the patients through the Walkon app was provided by Swallaby Co Ltd., the app’s developer. Walkon is a community-based app compatible with Android and iOS that promotes physical activities, particularly walking, to modify lifestyle habits and assist in managing underlying conditions [21]. Government agencies, companies, schools, and other institutions use it to promote walking [22]. The application collects user demographic information (eg, sex, age, height, and weight), as well as various activity data (eg, step count, sleep, types of activities, activity level, and calories). In addition, the company provides analyzed results to users and community administrators engaged in facilitating efficient health management [23]. The service also incorporates various gamification elements (eg, points and rewards) and social features, enabling users to increase their activity levels and manage their health in a more enjoyable and goal-oriented manner [22].

We collected clinical data from the Asan Medical Center, where the participants underwent regular examinations at the outpatient clinic. The patients visited the outpatient clinic during the baseline dates and 12 weeks, 6 months, and 12 months from the baseline. In breast cancer, patients were assessed for BMI and waist circumference to gather body measurement information. In addition, their blood sugar level, which levels were measured, including high-density lipoprotein (HDL) cholesterol, triglycerides, hemoglobin A_1c_ (HbA_1c_), and fasting blood sugar (FBS). Patients with colon cancer were examined to measure BMI, waist circumference for body measurements, metabolic parameters (HDL cholesterol, triglycerides, HbA_1c_, and FBS), and fat and muscle mass measurements that included skeletal muscle area, visceral fat (VFAT), and subcutaneous fat. For patients with lung cancer, the BMI was measured.

This study collected clinicopathologic data, such as age, sex, morbidities, tumor location, type of operation received, pathologic stage, and adjuvant treatments from the electronic medical records of the participants at the time of enrollment and every 6 months until the study concludes. At each outpatient clinic visit, the patient’s height and weight were measured using an electronic device, and the recorded values were directly entered into the electronic medical record, where the BMI was automatically calculated. Metabolic parameters (HDL cholesterol, triglycerides, HbA1c, and FBS) were also collected at each outpatient clinic, and FBS was measured after eight hours of fasting. For fat and muscle mass measured by abdominal computed tomography, an artificial intelligence software called AID-U (iAID) was used to select the third lumbar vertebra (L3) and assess body composition. A total of 2 experienced operators reviewed the quality of segmentation results in all L3 level segmentation images, and measurements were taken of the skeletal muscle area, including various muscles, as well as the visceral fat area and subcutaneous fat area.

In total, 2 surveys were used to evaluate the health-related quality of life: the HINT-8 questionnaire and the EQ-5D-5L. The HINT-8 consists of 8 items that assess various aspects, including climbing stairs, pain, vitality, work, depression, memory, sleep, and happiness. Each item is rated on a 4-point scale, indicating the level of problems experienced: no problems, mild, moderate, and severe problems. The HINT-8 allows for a comprehensive representation of 65,536 unique health states. On the other hand, the EQ-5D-5L comprises 5 factors: mobility, self-care, usual activities, pain and discomfort, and anxiety and depression. Each dimension is evaluated using a 5-point scale to describe the extent of problems: no problems, slight problems, moderate problems, severe problems, and extreme problems. In addition, the EuroQol VAS (Visual Analog Scale) is a rating system that ranges from 0 to 100, representing the worst to best imaginable health state, respectively. QOL questionnaires (HINT-8 and EQ-5D-5L) were conducted at both baseline and 6-month follow-up hospital visits.

### Data Analysis

#### Sample Entropy

We used the Sample Entropy module from the *antropy* package in Python (Python Software Foundation) to conduct the clustering of patient groups based on their step count over time. Sample entropy, a modified version of approximate entropy, is designed for analyzing physiological time-series signals [24]. It assesses the randomness of a series by directly using correlation integrals [25], offering advantages, including independence from data length and straightforward implementation, to make it more practical than approximate entropy [26]. To evaluate the contribution of app usage to regular physical activity, we used the Sample Entropy module to quantify the regularity or periodic trends of individual gait patterns. The entropy module was used based on the premise that, typically, the step count of patients tends to decrease over time following an intervention. This approach allows us to stratify patients into distinct groups based on their entropy levels, distinguishing between those with higher and lower levels of entropy. Hence, the classification process operates independently of the overarching trend of increasing or decreasing step counts. Once quantified, we can use k-means clustering to identify distinct clusters based on the gait patterns of patients [27]. The suitability of sample entropy for analyzing short and noisy biological datasets further enhances its applicability in this study [25]. Through comparing the sample entropy values, we classified patients into 2 clusters, namely the “active walking” and “inactive walking” groups, facilitating a more distinct interpretation of the obtained results. Sample entropy analysis was performed using Python (version 3.8.5).

#### Statistical Analysis

The 2 groups created based on step count underwent a Shapiro-Wilk test to assess the normality of their distributions. We conducted a paired *t* test to determine the statistical significance between parametric datasets and calculate their corresponding *P* values. Conversely, a Wilcoxon signed-rank test was used to calculate the *P* value for nonparametric datasets. We used the chi-square test to calculate the *P* values when analyzing survey items that used single-choice or yes or no responses. All data analyses were performed using R (version 4.3.0; R Foundation for Statistical Computing).

### Ethical Considerations

This study was approved by the Institutional Review Board of Asan Medical Center, Korea (IRB 2021-1631), and adhered to relevant ethical guidelines. Informed consent was obtained from all participants before their involvement in the study, with assurances of anonymity and confidentiality provided. Participants were briefed on the study’s objectives and how the collected data would be used. When obtaining patient consent, we explained that the log data of the smartphone application used for the intervention would be collected for research purposes in a pseudonymized form. In addition, stringent measures were in place to protect the privacy and confidentiality of study data, including secure storage within the hospital premises.

## Results

### Active Walking Group and Inactive Walking Group

**Figure 1:**
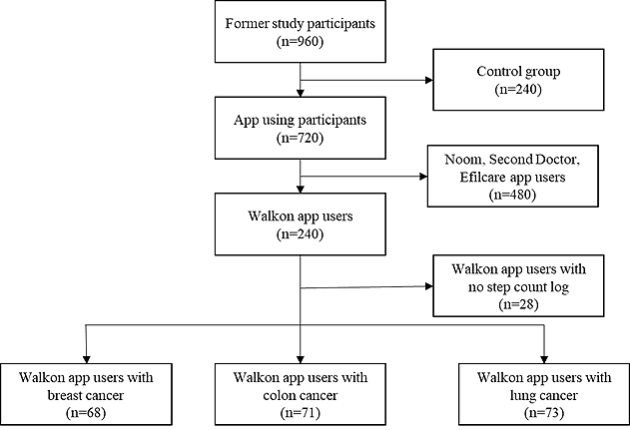
Flowchart of the patient selection process.

Out of 960 participants from the previous protocol study, 240 patients who used the Walkon application were selected, excluding patients in the control group or users of Noom (Noom Inc), Second Doctor (MediPlus Solution), and Efilcare (LifeSemantics Corp). We excluded 27 patients who did not have any step count logs recorded. The Walkon app had 212 users, consisting of patients with 3 types of cancer: breast, colon, and lung. There were 68 patients with breast cancer, 71 with colon cancer, and 73 with lung cancer. Figure 1 illustrates the patient selection process used in this study.

**Figure 2:**
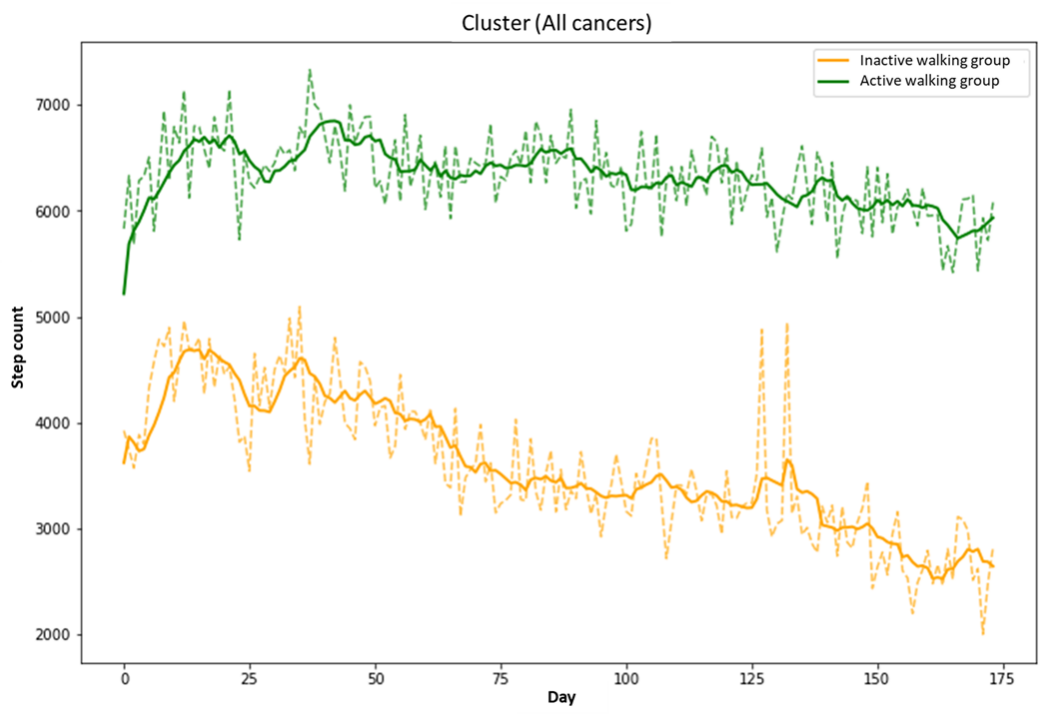
Clustering of time series of step counts (green line: active walking group; yellow line: inactive walking group).

We conducted 3 rounds of clustering to account for the varying availability of measurements across different types of cancer. The initial clustering included all patients (n=212) diagnosed with breast, colon, or lung cancer. Within this group, the active walking group (AWG) consisted of 137 patients, while the inactive walking group (IWG) comprised 75 patients. Figure 2 presents visual representations of the clustering results for each of these analyses.

Table 1 illustrates the disparities in median step counts between IWG and AWG. The median and mean daily step count were computed as the median and mean value of steps recorded on active days. The median and mean weekly and monthly step counts were determined by calculating the median and mean value of the total steps taken during the respective periods, divided by the duration of each period. In terms of daily step count, the IWG exhibited a median of 4223 (IQR 1497-8446) steps and a mean of 5660 (SD 5523.9), while the AWG recorded 5355 (IQR 2326-9289) steps and a mean of 6310 (SD 4973.6). Moving to weekly step counts, the IWG displayed a median of 13887 (IQR 5827-34,437) and a mean of 24,480 (SD 25,535.4), whereas the AWG demonstrated a median of 40,247 (IQR 27,414-52,561) and a mean of 42,840 (SD 22,639). Finally, the median monthly step counts were 60,178 (IQR 25,446-149,225) and 174,405 (IQR 118,794-227,765) for the IWG and AWG, respectively, with mean monthly step counts of 106,083 (SD 110,654) and 185,642 (SD 98,102.4).

**Table 1:** Step count, median (IQR) of each group.

	Inactive walking group (n=75)	Active walking group (n=137)
Daily step count, median (IQR)	4223 (1497-8446)	5355 (2326-9289)
Weekly step count, median (IQR)	13,887 (5872-34,437)	40,247 (27,414-52,561)
Monthly step count, median (IQR)	60,178 (25,446-149,225)	174,405 (118,794-227,765)

### Characteristics of the Active and Inactive Walking Groups

For 212 Walkon app users, we used the clustering technique, specifically sample entropy. We classified these patients into 2 distinct groups: the AWG and the IWG, comprising 137 and 75 patients, respectively. Table 2 lists the general characteristics of the 212 patients, categorized according to their respective groups. No significant differences were observed in demographic factors, such as body measurements, cancer stage, chemotherapy status, education status, marital status, religion, and monthly family income between the two groups. However, statistically significant differences were noted in terms of sex, age, and cancer type between the AWG and the IWG.

**Table 2:** Demographic characteristics of participants.

Characteristics		Total (N=212)	Inactive walking group (n=75)	Active walking group (n=137)	*P* value	
**Sex, n (%)**						.01
	Male	78 (36.8)	37 (49.3)	41 (29.9)		
	Female	134 (63.2)	38 (50.7)	96 (70.1)		
Age (years), mean (SD)		55.3 (10.1)	58 (9.5)	53.8 (10.1)	.003	
**Body measurements, mean (SD)**						
	Weight (kg)	63.5 (11.2)	63.9 (10.2)	63.2 (11.8)	.56	
	BMI (kg/m^2^)	24 (3.4)	24 (2.9)	24 (3.6)	.96	
**Cancer type, n (%)**						.01
	Breast	68 (32.1)	15 (20)	53 (38.7)		
	Colon	71 (33.5)	34 (45.3)	37 (27)		
	Lung	73 (34.4)	26 (34.7)	47 (34.3)		
**Cancer stage, n (%)**						.06
	0	8 (3.8)	0 (0)	8 (5.8)		
	I	111 (52.4)	38 (50.7)	73 (53.3)		
	II	46 (21.7)	14 (18.7)	32 (23.4)		
	III	47 (22.2)	23 (30.7)	24 (17.5)		
**Chemotherapy, n (%)**						.25
	Yes	69 (32.5)	29 (38.7)	40 (29.2)		
	No	143 (67.5)	46 (61.3)	97 (70.8)		
**Education, n (%)**						.11
	Middle school or lower	16 (7.5)	7 (9.3)	9 (6.6)		
	High school	82 (38.7)	35 (46.7)	47 (34.3)		
	Bachelor’s degree or higher	114 (53.8)	33 (44)	81 (59.1)		
**Marital status, n (%)**						.1
	Married	184 (86.8)	61 (81.3)	123 (89.8)		
	Not married	18 (8.5)	7 (9.3)	11 (8)		
	Divorced	8 (3.8)	6 (8)	2 (1.5)		
	Bereaved	2 (0.9)	1 (1.3)	1 (0.7)		
**Religion, n (%)**						.47
	None	94 (44.3)	35 (46.7)	59 (43.1)		
	Buddhist	52 (24.5)	16 (21.3)	36 (26.3)		
	Catholic	46 (21.7)	14 (18.7)	32 (23.4)		
	Christian	19 (9)	10 (13.3)	9 (6.6)		
	Other	1 (0.5)	0 (0)	1 (0.7)		
**Monthly family income (US $)^a^, n (%)**						.54
	Less than 1500	26 (12.3)	11 (14.7)	15 (10.9)		
	1500-3000	70 (33)	28 (37.3)	42 (30.7)		
	3000-4500	64 (30.2)	20 (26.7)	44 (32.1)		
	More than 4500	52 (24.5)	16 (21.3)	36 (26.3)		

^a^The exchange rate calculation is based on the initial data analysis period of June-July 2023, and US $1 was calculated as 1300 won.

### Biometric Data Comparison

Table 3 presents the impact of walking activity on body measurements, blood sugar levels, and body composition for 2 groups: the AWG and IWG. HDL cholesterol and triglyceride levels showed statistically significant differences between baseline and 6 months for both the AWG and the IWG. In addition, the AWG exhibited statistically significant differences in VFAT and waist circumference after 6 months. However, the remaining measurements, including BMI, HbA_1c_, FBS, skeletal muscle area, and subcutaneous fat, did not exhibit a statistically significant difference after 6 months. Furthermore, no statistically significant differences were observed between the IWG and the AWG in any of the measurements. Table S1 in Multimedia Appendix 1 shows the impact of walking levels on the changes in body measurements, blood sugar level, and body composition between baseline and 12-month follow-up. While some measurements showed differences at the 12-month follow-up, none of these differences were statistically significant between the groups.

**Table 3:** Baseline and 6-month changes in various measurements for inactive and active walking groups.

Metabolic and body composition indicators		Inactive walking group				Active walking group				*P* value^a^
		Baseline	6 months	*P* value	Baseline		6 months	*P* value		
**Body measurements, mean (SD)**										
	BMI^b^	23.34 (2.9)	23.4 (3)	.74	24.14 (3.8)		23.81 (3.8)	.25	.65	
	Waist^c^	79.13 (7.7)	78.28 (7.9)	.58	79.02 (8.6)		77.16 (9.8)	.01	.26	
**Blood sugar^c^, mean (SD)**										
	HDL^d^ cholesterol	42.39 (9.5)	52.61 (13.5)	<.001	46.35 (12.9)		57.32 (13.7)	<.001	.84	
	Triglyceride	102.25 (33.9)	133.16 (65.8)	.02	102.04 (43.5)		129.15 (87.5)	<.001	.67	
	HbA_1c_	5.65 (0.5)	5.87 (1.4)	.84	5.67 (0.9)		5.75 (1.0)	.13	.45	
	FBS^e^	114.50 (20.8)	123.39 (85.6)	.09	111.24 (23.8)		111.78 (38.4)	.51	.30	
**Abdominal CT^f,g^, mean (SD)**										
	SMA^h^	137.33 (25.8)	134.25 (24.7)	.31	128.46 (32.6)		128.12 (31.5)	.85	.31	
	SFAT^i^	138.29 (44.3)	139.71 (50.2)	.66	148.85 (71.8)		148.28 (74.0)	.86	.84	
	VFAT^j^	141.06 (66.9)	133.77 (62.1)	.70	122.45 (64.8)		100.98 (50.8)	.002	.46	

^a^Significance of the delta (change) between the active and inactive walking groups.

^b^Breast, colon, and lung cancer (n=212): Group 1 (n=75) + Group 2 (n=137).

^c^Breast and colon cancer (n=139): Group 1 (n=47) + Group 2 (n=92).

^d^HDL: high-density lipoprotein.

^e^FBS: fasting blood sugar.

^f^Colon cancer (n=72): Group 1 (n=25) + Group 2 (n=47).

^g^CT: computed tomography.

^h^SMA: skeletal muscle area.

^i^SFAT: subcutaneous fat area.

^j^VFAT: visceral fat area.

### QOL Comparison

Table 4 illustrates the disparities between the IWG and AWG concerning QOL questionnaires (EQ-5D-5L and HINT-8). Both the IWG and AWG showed significant improvements in the domains of movement, self, activity, pain, and VAS. However, only the VAS exhibited a significant difference between the AWG and IWG. Conversely, within the HINT-8 questionnaire, pain emerged as the sole factor displaying a significant difference between the two groups.

**Table 4:** Baseline and 6-month changes in quality of life questionnaires for inactive and active walking groups.

Questionnaire			Inactive walking group (n=75)							Active walking group (n=137)							
			Baseline		6 months		*P* value		Baseline			6 months		*P* value		*P* value^a^	
**EQ-5D-5L^b,c^, mean (SD)**																	
	Move	1.75 (0.82)		1.24 (0.65)		*<.001^d^*		1.53 (0.8)			1.13 (0.45)		*<.001*		.16		
	Self	1.69 (0.87)		1.06 (0.24)		*<.001*		1.67 (0.73)			1.04 (0.3)		*<.001*		.86		
	Activity	1.91 (0.93)		1.25 (0.58)		*<.001*		1.92 (0.84)			1.16 (0.43)		*<.001*		.58		
	Pain	2.29 (0.77)		1.72 (0.62)		*<.001*		2.18 (0.69)			1.56 (0.63)		*<.001*		.61		
	Anxiety	1.73 (0.7)		1.74 (0.75)		.99		1.68 (0.72)			1.61 (0.69)		.47		.64		
	Visual Analog Scale	65.93 (18.95)		72.94 (20.54)		.004		64.03 (18.4)			79.25 (13.96)		*<.001*		*.03*		
**HINT-8^e,f^, mean (SD)**																	
	Climb	1.65 (0.73)		1.47 (0.63)		.11		1.51 (0.71)			1.36 (0.55)		.11		.56		
	Pain	1.75 (0.68)		1.38 (0.49)		*<.001*		1.54 (0.63)			1.48 (0.54)		.57		*.01*		
	Energy	2.07 (0.79)		2.07 (0.70)		.89		2.01 (0.79)			1.8 (0.67)		*.01*		.14		
	Work	1.59 (0.64)		1.53 (0.68)		.53		1.57 (0.66)			1.35 (0.52)		.003		.29		
	Depression	1.73 (0.62)		1.81 (0.65)		.43		1.71 (0.64)			1.57 (0.61)		*.02*		.11		
	Memory	1.41 (0.5)		1.49 (0.5)		.31		1.34 (0.49)			1.48 (0.54)		*.01*		.59		
	Sleep	1.83 (0.69)		1.59 (0.74)		*.01*		1.7 (0.71)			1.55 (0.68)		*.04*		.18		
	Happiness	2.19 (0.71)		2.29 (0.79)		.36		2.20 (0.72)			2.08 (0.64)		*.05*		.06		

^a^Significance of the delta (change) between the active and inactive walking groups.

^b^EQ-5D-5L: European Quality of Life 5 Dimensions 5 Level version.

^c^EQ-5D-5L is measured across five levels; 1=no problems, 2=indicates slight problems, 3=moderate problems, 4=severe problems, and 5=extreme problems or inability to perform the activity.

^d^Values in italics emphasize statistical significance for *P* values <.05.

^e^HINT-8: Health-related Quality of Life Instrument with 8 Items.

^f^HINT-8 is measured across four levels: 1=no problems, 2=mild problems, 3=moderate problems, and 4=severe problems.

## Discussion

### Principal Findings

In this study, we successfully classified patients according to their step count patterns, ensuring distinct groups without any overlap throughout the entire time span. The distinct classification implies that periodic walking habits, logged and monitored by the app, are correlated with the amount of physical activity. It further demonstrated the strength and scalability of our methodology in categorizing patients according to their walking habits.

The effectiveness of this classification became apparent as the group displaying greater physical activity predominantly included female patients and younger individuals, which is consistent with previous research findings [7,28,29]. Therefore, our method not only mitigated potential confounding factors but also highlighted the effectiveness of our classification approach. Across all 3 clustering rounds, the AWG consistently demonstrated a prolonged and stable walking pattern. Conversely, the IWG exhibited a significant decrease in step count over time, which was particularly evident when monitoring measurements up to the 6-month mark. Physical activity management apps, such as Walkon, aim to promote continuous behavioral change (habituation) rather than seeking immediate responses. Therefore, this classification result is considered appropriate.

This study did not find a significant difference in biometric data changes between the baseline and the 6-month follow-up between the AWG and IWG. However, significant differences were observed in pain and the VAS in relation to QOL questionnaires. Especially for pain, at baseline, the mean value for patients was 1.75, which is closer to 2, indicating that patients reported weak pain. However, at 6 months, the value became closer to 1 (1.38), indicating a lack of pain and, thus, a clinical improvement. Nevertheless, regarding biometric measurements, only waist circumference and visceral fat indicated substantial improvements, specifically within the AWG. In addition, with respect to the QOL questionnaire, improvements in energy, work, depression, memory, and happiness were statistically significant only in the AWG, whereas other factors did not show significant differences.

### Comparison With Previous Studies

The IWG and AWG showed significant differences in demographic factors such as sex, age, and cancer types. At the same time, the cancer stage did not exhibit a significant difference between AWG and IWG, where patients with lower cancer stages appeared to be more prevalent in the AWG than in the IWG. This variation could be due to the different average ages at which each type of cancer was diagnosed. Patients with breast cancer had the highest number of diagnoses in their late 40s, but colon cancer and lung cancer tended to be most commonly diagnosed in the early 60s and 70s, respectively [30]. However, from another perspective, we can observe the effectiveness of the current methodology in distinguishing patients by aligning with preliminary studies. In other studies, the group that walked regularly, making walking a habitual activity, consisted predominantly of young female patients, regardless of their disease [28,29].

Regarding the comparison of biometric data between AWG and IWG, no variables demonstrated a significant difference in the extent of change between the baseline and the 6-month follow-up. Nevertheless, both walking groups showed significant improvement in HDL cholesterol. This may be due to the amount of activity, but it cannot be ruled out that it may also be due to lipid metabolism abnormalities caused by chemotherapy [31-33]. Compared with the baseline, waist circumference and BMI decreased, but most of the blood test results worsened. This decline is likely due to reduced physical activity as a result of cancer treatment [7]. Remarkably, the AWG consistently displayed a healthier profile at the 6-month follow-up in both of these factors compared with the IWG. Furthermore, while waist circumference was comparable between the AWG and IWG at baseline, the AWG group exhibited a significant decrease after 6 months. Similarly, in terms of VFAT, although the difference between the AWG and IWG was not statistically significant, the mean VFAT value for AWG experienced a significant reduction, indicating improvement.

Even though Backman and Wengström [34] demonstrated that enhanced physical activity, such as daily walks of 10,000 steps over a 10-week period, results in improved breast symptoms and decreased weight gain for patients with breast cancer undergoing adjuvant chemotherapy, our study did not show a decrease in weight gain during the follow-up period. Although no significant differences in biometric factors between the AWG and IWG were observed after the 6-month follow-up, certain factors showed significant improvements only within the AWG. This suggests that maintaining a steady, high level of walking could potentially yield greater health benefits compared with a group with less consistent and lower walking activity.

In the QOL questionnaires, the pain factor from HINT-8 showed a significant difference in pain perception. Pain showed greater improvement in the IWG than in the AWG. Since studies have reported nonserious adverse events like joint pain or back pain in patients with cancer undergoing physical activity interventions [35], the observed increase in pain among the participants in the AWG could potentially be attributed to these factors. In addition, considering that the AWG started with lower baseline pain scores than the IWG, there is a possibility that individuals who initially had less pain might have found it easier to improve their time walking ability. Since the questionnaire inquired about general pain without specifying a particular ailment, it is challenging to ascertain the specific type of pain to which the patients are referring.

However, despite a similar baseline value, there was a more pronounced improvement observed in the AWG in terms of the VAS, as against the EQ-5D-5L, during the 6-month follow-up period. This finding, aligned with the VAS, yielded significant results in all models across various types of cancer, including breast, lung, and prostate cancers, in artificial intelligence health trials [36,37]. As an outcome variable of habit change, VAS demonstrated the effectiveness of VAS as a sensitive, intuitive, and patient-subjective comprehensive score in assessing the current state of a patient. Surprisingly, despite experiencing more pain, patients in the AWG reported an improved state of well-being compared with the IWG. Although stress generally has a negative effect on physical activity, varying results were reported in association with differing study designs and sources of stress [38]. Therefore, we need to consider the properties of the questionnaire used to measure QoL from various perspectives.

In addition, although the difference between AWG and IWG was not significant, AWG showed a significant improvement in the happiness questionnaire, while IWG showed a deterioration. Although a significant change was not evident in most factors between the AWG and IWG, the 6-month follow-up mean scores for factors from EQ-5D-5L consistently demonstrated a more favorable status in the AWG compared with the IWG. In addition, within the HINT-8 questionnaire, apart from pain, all other factors exhibited a more substantial improvement in mean values within the AWG when compared with the IWG. The present findings indicate that while there may be discernible changes in QOL following digital intervention, their significance is not strong. Notably, Kim et al [39] highlighted the potential of home-based exercise programs in enhancing QOL among colorectal cancer survivors. However, contrasting results were reported by Dhillon et al [40], who explored the impact of physical activity on QOL among advanced lung patients with cancer, where it revealed no significant improvement during 2-, 4-, and 6-month follow-up periods [40].

Most studies that have examined the effect of physical activity on patients with cancer included an additional exercise program that included supervised exercise sessions with an exercise specialist and exercise videos [34,38,41]. In our study, motivation to increase step counts was solely derived from the Walkon app. Del Pozo Cruz et al [42] and Paluch et al [43] have shown that taking up to 10,000 steps per day, or between 6000 to 10,000 steps depending on age, may lower the risk of mortality and cancer. However, both the AWG and IWG had a daily step count lower than 6000, which may be considered low compared with the levels suggested by other studies.

### Strength and Limitations

Numerous studies have investigated the effect of increased levels of physical activity through digital intervention [44]. However, to the best of our knowledge, we are the first study that has examined the step count trends of patients with a digital intervention and reviewed the intergroup differences in various health outcomes and quality of life parameters. In addition, we have successfully introduced a methodology that incorporated periodic walking habits, while the majority of the research used simple calculations to measure step count [45,46]. It is expected that this method will be able to evaluate behavioral changes intended by many physical activity interventions in a much more quantitative, objective, and reproducible manner than existing subjective reports.

Furthermore, we believe we are the first study to investigate all blood test results, body compositions, and QOL questionnaires to study the effect of walking levels on patients with cancer. In addition, one of the strengths of this study is that it includes both 6-month and 12-month follow-up periods, qualifying it as a long-term investigation; previous randomized controlled trials that examined the efficacy of computer-tailored physical activity interventions have often lacked extended follow-up periods [47]. Furthermore, a controversy exists regarding the use of BMI as a simple method to demonstrate outcomes despite its limitations in effectively assessing body composition [48]. To address this issue, we have incorporated BMI results alongside body composition measurements. This inclusion enables a more comprehensive understanding of how interventions influence the body.

However, this study has some limitations. First, we observed that the AWG includes a higher proportion of patients with breast cancer who are predominantly female and relatively younger. This demographic difference could potentially have amplified the variations in responses. Nonetheless, the inclusion of patients with breast cancer was essential to assess the broader impact of step count trends on quality of life among patients with cancer. However, the diversity in cancer types, especially the high prevalence of breast cancers and a notable representation of younger participants within our analyzed cohort, may diminish the generalizability of the observed impact. However, this result could paradoxically underscore the effectiveness of our meticulous rate-gazing and clustering methodology. It is noteworthy that existing literature consistently highlights that habitual walkers predominantly consist of women and younger individuals. Thus, this discovery potentially validates our demographic differentiation approach as an effective strategy rather than merely amplifying the variance in responses.

Second, for iPhone users, the app had to be activated to retrieve data from the phone, which could have resulted in unregistered step counts when the app was not running. Third, since all participants were of Korean ethnicity, the study could not account for racial variations. Finally, since these questionnaires and measurements were designed to assess the fragility of patients with cancer throughout the disease trajectory, it is worth noting that they have limitations when it comes to evaluating patient fitness. Hence, using measurements specifically designed to evaluate patient fitness would be a more appropriate course of action. Finally, this study does not have a sufficient follow-up period to observe cancer outcomes such as mortality. Therefore, it serves as a preliminary study that will be followed by a more comprehensive follow-up study later.

### Implications and Future Research

This study’s methodology for evaluating and categorizing patients based on their step count data could potentially be extended to analyze other types of time series data. The rigorous methodology used here may serve as a model for categorizing patients based on various types of longitudinal data in future research endeavors. For future investigations, the inclusion of dietary-related content could provide valuable insights, as this study solely incorporated body measurements and QOL questionnaires. Furthermore, we propose that a study concentrating on long-term survival and mortality would significantly enhance the value of this research in the future. As the Walkon app can be offered to any patient, populations other than those with cancer can be investigated to assess the generalizability of the app’s impact.

### Conclusion

Through this study, we effectively categorized patients based on their step count trends, ensuring distinct groups without overlap throughout the time series. Our analysis revealed clear quantitative differences in step counts, demonstrating the robustness and scalability of our methodology in categorizing patients based on their step count log data. Importantly, the success of this categorization was evident as the group with higher levels of physical activity mainly consisted of patients with breast cancer and young individuals, which is consistent with findings from previous literature. Thus, our approach not only avoided confounding effects but also highlighted the effectiveness of our categorization strategy. However, while this study revealed significant improvements within the AWG, there were instances where the observed enhancements did not reach statistical significance when compared with the IWG. Notably, only 2 factors exhibited statistically significant differences: pain and the VAS factor from the QOL questionnaire. Interestingly, pain exhibited a more prominent improvement within the IWG, whereas the VAS factor still displayed more improvement within the AWG. In the realm of biometric data, no factors displayed statistically significant disparities between the AWG and IWG. However, waist circumference and visceral fat showed significant improvements only in the AWG. Furthermore, within the sphere of QOL, only the AWG demonstrated significant improvements in energy, work, depression, memory, and happiness. This suggests that AWG, characterized by steady and slightly augmented levels of physical activity, tends to result in significant improvements in health outcomes. Hence, it seems essential for patients with cancer to engage in regular walking exercises.

## Data Availability

The datasets generated or analyzed during this study are not publicly available in accordance with the data sharing policy of the institutional review board of Asan Medical Center (Seoul, Republic of Korea) but are available from the corresponding author on reasonable request.

## Appendix

Multimedia Appendix 1 Changes between baseline and 12 months for various measurements for inactive walking group and active walking group.

## Abbreviations

AWG: active walking group
EQ-5D-5L: European Quality of Life 5 Dimensions 5 Level version
FBS: fasting blood sugar
HbA1c: hemoglobin A1c
HDL: high-density lipoprotein
HINT-8: Health-related Quality of Life Instrument with 8 Items
IWG: inactive walking group
QOL: quality of life
VAS: Visual Analog Scale
VFAT: visceral fat
