# Supplementary material for: Analysis of Metabolic and Quality-of-Life Factors in Patients With Cancer for a New Approach to Classifying Walking Habits: Secondary Analysis of a Randomized Controlled Trial
Source: J Med Internet Res. 2025 Apr 1;27:e52694. doi: 10.2196/52694 (PMC12000789; doi:10.2196/52694)
Supplement: Multimedia Appendix 1 [file jmir_v27i1e52694_app1.docx]

**Table S1. Changes between baseline and 12 months for various measurements for inactive walking group and active walking group.**

|  | **Group 1** | | |  | **Group 2** | | | **p-value (delta)** |
| --- | --- | --- | --- | --- | --- | --- | --- | --- |
|  | **baseline** | **12months** | **p-value** |  | **baseline** | **12months** | **p-value** |  |
|  | **mean (std)** | |  |  | **mean (std)** | |  |  |
| Body measurements |  |  |  |  |  |  |  |  |
| BMI^a^ | 23.34 (2.9) | 23.61 (2.9) | 0.77 |  | 24.14 (3.8) | 24.10 (4.1) | 0.93 | 0.49 |
| Waist^b^ | 79.13 (7.7) | 78.67 (8.5) | 0.35 |  | 79.02 (8.6) | 78.43 (9.9) | 0.75 | 0.71 |
|  |  |  |  |  |  |  |  |  |
| Blood sugar^b^ |  |  |  |  |  |  |  |  |
| HDL cholesterol | 42.39 (9.5) | 54.17 (12.1) | **0.00** |  | 46.35 (12.9) | 58.66 (14.7) | **0.00** | 0.85 |
| Triglyceride | 102.25 (33.9) | 107.29 (52.3) | 0.61 |  | 102.04 (43.5) | 125.20 (73.5) | **0.00** | 0.06 |
| HbA1c | 5.65 (0.5) | 5.71 (1.0) | 0.95 |  | 5.67 (0.9) | 5.71 (0.8) | 0.02 | 0.14 |
| FBS | 114.50 (20.8) | 106.88 (28.5) | **0.00** |  | 111.24 (23.8) | 104.42 (18.8) | **0.03** | 0.13 |

^a^Breast, Colon, and Lung cancer (n=212): Group1 (n=75) + Group2 (n=137).

^b^Breast and Colon cancer (n=139): Group1 (n=47) + Group2 (n=92).
